# LDLR-Gene therapy for familial hypercholesterolaemia: problems, progress, and perspectives

**DOI:** 10.1186/1755-7682-3-36

**Published:** 2010-12-13

**Authors:** Faisal A Al-Allaf, Charles Coutelle, Simon N Waddington, Anna L David, Richard Harbottle, Michael Themis

**Affiliations:** 1 Department of Medical Genetics, Faculty of Medicine, Umm Al-Qura University, Al-Abedia Campus, P. O. Box 715, Makkah 21955, Saudi Arabia; 2 Gene Therapy Research Group, Department of Molecular and Cell Medicine, Sir Alexander Fleming Building, Faculty of Medicine, Imperial College London, London SW7 2AZ, UK; 3 Department of Haematology, Haemophilia Centre and Haemostasis Unit, Royal Free and University College Medical School, London NW3 2PF, UK; 4 Prenatal Cell and Gene Therapy Group, Institute for Women's Health, University College London, 86 - 96 Chenies Mews, London, WC1E 6HX, UK; 5 Gene Therapy and Genotoxicity Research Group, Brunel University, Heinz Wolff Building, Uxbridge, Middlesex, West London UB8 3PH, UK

## Abstract

Coronary artery diseases (CAD) inflict a heavy economical and social burden on most populations and contribute significantly to their morbidity and mortality rates. Low-density lipoprotein receptor (LDLR) associated familial hypercholesterolemia (FH) is the most frequent Mendelian disorder and is a major risk factor for the development of CAD. To date there is no cure for FH. The primary goal of clinical management is to control hypercholesterolaemia in order to decrease the risk of atherosclerosis and to prevent CAD. Permanent phenotypic correction with single administration of a gene therapeutic vector is a goal still needing to be achieved. The first *ex vivo *clinical trial of gene therapy in FH was conducted nearly 18 years ago. Patients who had inherited LDLR gene mutations were subjected to an aggressive surgical intervention involving partial hepatectomy to obtain the patient's own hepatocytes for *ex vivo *gene transfer with a replication deficient LDLR-retroviral vector. After successful re-infusion of transduced cells through a catheter placed in the inferior mesenteric vein at the time of liver resection, only low-level expression of the transferred LDLR gene was observed in the five patients enrolled in the trial. In contrast, full reversal of hypercholesterolaemia was later demonstrated in *in vivo *preclinical studies using LDLR-adenovirus mediated gene transfer. However, the high efficiency of cell division independent gene transfer by adenovirus vectors is limited by their short-term persistence due to episomal maintenance and the cytotoxicity of these highly immunogenic viruses. Novel long-term persisting vectors derived from adeno-associated viruses and lentiviruses, are now available and investigations are underway to determine their safety and efficiency in preparation for clinical application for a variety of diseases. Several novel non-viral based therapies have also been developed recently to lower LDL-C serum levels in FH patients. This article reviews the progress made in the 18 years since the first clinical trial for gene therapy of FH, with emphasis on the development, design, performance and limitations of viral based gene transfer vectors used in studies to ameliorate the effects of LDLR deficiency.

## Introduction

Familial hypercholesterolaemia (FH) is primarily an autosomal dominant disorder, characterised by a lifelong elevation of serum cholesterol bound to low-density lipoprotein (LDL). The primary causative defects in approximately 85% of FH cases are mutations or deletions in the plasma membrane Low Density Lipoprotein Receptor (LDLR) encoding gene that is responsible for clearing LDL-cholesterol (LDL-C) from the blood stream by endocytosis and intracellular degradation [1]. Over 1000 different mutations in the LDLR gene on the distal short arm of chromosome 19 (p13.1-p13.3) have been described to date [2] and are recorded online at http://www.ucl.ac.uk/ldlr/Current/[3]. The second gene responsible for fewer than 10% of FH cases encodes the ligand for LDLR, namely Apolipoprotein B-100 (ApoB-100), located on the short arm of chromosome 2 (p24) [4]. Mutations in this gene reduce ligand affinity for the receptors and cause reduced clearance of LDL particles resulting in hypercholesterolemia [5], albeit normal LDLR activity. A mutation in the codon for amino acid 3500 (CGG-to-CAG) was found to be a CG mutation hotspot associated with defective LDLs and hypercholesterolemia [6]. The pathophysiological consequences from LDLR or ApoB mutations are loss of protein function, which lead to monogenic FH. Defects in a third gene, located on the short arm of chromosome 1 (p34.1-p32), have also been identified to cause monogenic FH [7]. The convertase subtilisin/kexin type 9 (PCSK9)-gene codes for an enzyme that has also been called ''neural apoptosis regulated convertase 1'', which has been proposed to be involved in degrading the LDLR protein in the lysosome and thus preventing it from recycling [8]. Gain of function mutations in the PCSK9 gene could therefore cause increased degradation of LDLRs, reduced numbers of receptors on the surface of the cell, and monogenic FH. An autosomal recessive form of FH caused by loss of function mutations in the LDLRAP1 gene, which is located on the short arm of chromosome 1p35-36.1, has also been documented [9]. The clinical phenotype of the autosomal recessive form is similar to that of the classic homozygous FH caused by defects in the LDLR gene, but it is generally less severe and more responsive to lipid-lowering therapy (reviewed in [10]). This article focuses on LDLR-associated FH reviewing, the encountered obstacles, the achieved progress and the future prospectives of LDLR-gene therapy for this disease.

## LDLR-associated FH

Owing to mutations in both alleles of the LDLR locus, homozygous LDLR-associated FH patients present with markedly elevated total serum cholesterol (>500 mg/dL, 13 mmol/L) and LDL-cholesterol levels (LDL-C, >450 mg/dL, 11.7 mmol/L). The deposition of insoluble cholesterol causes xanthomata on the tendons of the hands and feet, cutaneous planar and corneal arcus in early life [11,12]. Atheroma of the aortic root and valve can lead to myocardial infarction (MI) and sudden death before the age of 30 years. Coronary artery disease (CAD) is more common and more extensive in receptor negative patients (mutations that completely eliminate receptor functions) than in those with the receptor-defective type (mutations that partially inactivate receptor function), where there is residual receptor activity [12,13]. Heterozygous patients typically have a lower serum cholesterol level (250-450 mg/dL or 6.5-11.6 mmol/L) and LDL-C (200-400 mg/dL or 5.2-10.4 mmol/L) with positive age correlation. They develop the above clinical features at a less accelerated rate, but if untreated most suffer a severe MI and often sudden death or other cardiovascular events in the fourth or fifth decade of life. Due to several hormonal factors, approximately 80% of heterozygote men suffer from CAD, while only 20% to 30% of women are moderately affected [14].

In most investigated populations, the heterozygote form occurs in at least 1:500 and the homozygous form in one in one million individuals [15], although in some populations, for example the Afrikaner population in South Africa, heterozygosity is found in less than 1:80 individuals [16,17]. This unusual high frequency is due to founder effects and no heterozygote advantage has been identified. Heterozygous FH is therefore the most frequent clinically relevant Mendelian trait, being more frequent than homozygous cystic fibrosis and sickle cell anaemia.

Cholesterol levels alone are not sufficient to confirm a diagnosis of FH because blood cholesterol levels vary with age, gender and are population specific [18]. In addition, the range of blood cholesterol levels in FH overlaps with that of people with non-genetic multifactorial hypercholesterolaemia, which reduces diagnostic accuracy. Diagnostic criteria of FH, therefore, include clinical symptoms and laboratory findings as well as the family history of a dominant pattern of inheritance for either premature coronary heart disease or hypercholesterolaemia, (reviewed in [18]).

The human LDLR is a multi-component single-chain glycoprotein, which contains 839 amino acids in its mature form, encoded by a gene of 45 kb in length [19]. The gene contains 18 exons of which 13 exons code for protein sequences that show homology to other proteins such as the C9 component [20], Epidermal Growth Factor (EGF) [21], blood coagulation factor IX, factor X (FX) and protein C [22-24]. The mRNA transcript is 5.3 kb in length and encodes a protein of 860 amino acids. About half of the mRNA constitutes a long 3' untranslated region that contains two and a half copies of the *Alu *family of middle repetitive DNAs [25]. LDL-Receptors are expressed ubiquitously by almost all somatic cells under control of sterol negative feedback, mediated by three 16 bp imperfect repeats (sterol regulatory elements) and a TATA box like sequence in the promoter [26]. Their function is to bind to apolipoprotein ligands, apoB-100 and apoE. Uptake of LDL is mediated mainly through apoB-100 [27].

**Figure 1:**
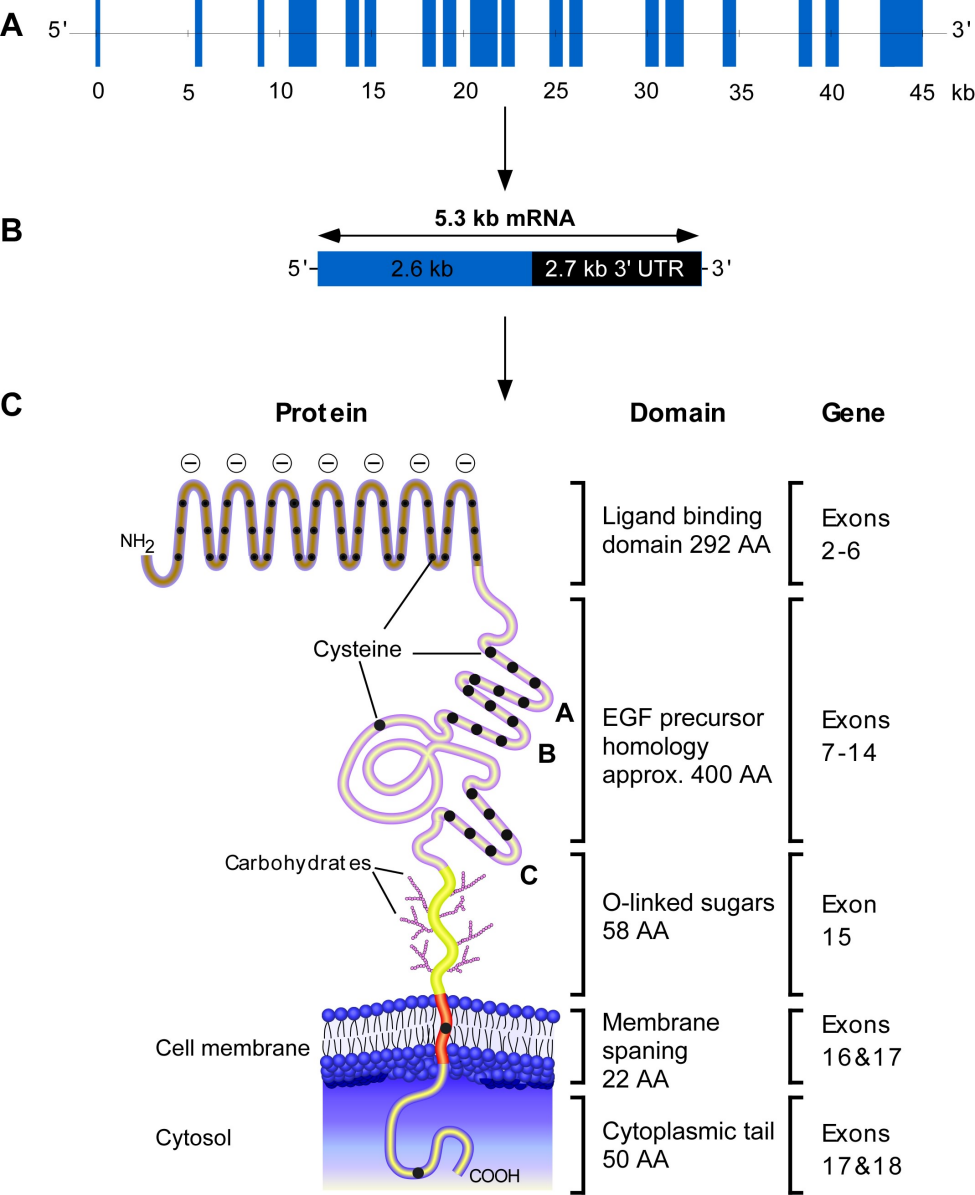
**Schematic representation of the human LDLR gene, mRNA and protein, A, B and C, respectively. UTR, untranslated region of the mRNA transcript. **Reproduced with modifications from[25].

The mature human LDLR of 160 kDa is composed of five domains, Figure 1. Exon 1 encodes a short 5' untranslated region and 21 hydrophobic amino acids that are not present in the mature protein. This sequence functions as a signal peptide to direct the receptor synthesising ribosomes to the Endoplasmic Reticulum (ER) membrane [25]. Other functional domains of the peptide correspond to the exons as indicated in Figure 1, 41 bp of exon 17 plus exon 16 encode the transmembrane domain and the reminder of exon 17 together with exon 18 encode the cytoplasmic domain.

Analyses of LDLR-associated FH variants estimated that there were 1066 LDLR gene mutations/rearrangements, 65% (n = 689) of which were DNA substitutions, 24% (n = 260) small DNA rearrangements (<100 bp), and 11% (n = 117) large DNA rearrangements (>100 bp) [2]. The DNA substitutions and small rearrangements occur along the length of the gene, with 839 in the exons (93 nonsense variants, 499 missense variants and 247 small rearrangements), 86 in intronic sequences, and 24 in the promoter region. The highest proportion of exon variants occurs in the ligand binding domain (exons 2-6) and the EGF precursor domain (exons 7-14) [2].

## Clinical management of FH

To date there is no cure for FH. The primary goal of clinical management in heterozygotes is to control hypercholesterolaemia by lifestyle modification and/or drug treatment in order to decrease the risk of atherosclerosis and to prevent CAD. Lifestyle modification involves educating patients to adhere to a low-fat diet, exercise and to reduce overweight or maintain an optimal body weight. An effective low-fat diet could lower LDL-C (LDL cholesterol) by 20% to 30% [28-30]. For patients who are not able to reach their LDL-C goal (<129 mg/dI, 3.31 mmol/L) on the lifestyle modification program, drug therapy is the next step. The current recommendations for LDL-C goals from the National Cholesterol Education Program Adult Treatment Panel III guidelines are <100 mg/dI, 2.586 mmol/L for patients with very high cardiovascular risk and <129 mg/dI, 3.31 mmol/L for patients with moderate cardiovascular risk [31]. The preferred and most effective lipid-lowering agents are the 3-hydroxy-3-methylglutaryl-coenzyme A (HMG-CoA) reductase inhibitors, more commonly known as statins [32]. Statins are the best tolerated medication in patients of all ethnic groups, both sexes, and generally, all ages. They also have an excellent safety profile over the now nearly 20 years of widespread clinical use, and have the highest level of patient adherence among available lipid-lowering agents with low incidence of side effects [33]. Because different statins have variable potency, the therapeutic outcome ranging from 20% to 60% reduction in LDL-C [32], depends on the particular statin used, the dose and the type of LDLR mutation. Despite the powerful effect of statins, they may not be appropriate for those who are best treated with non-systemic therapy (eg, young adults, women of childbearing age), who require only a modest reduction in LDL-C, or those with active liver disease or increased liver function test values and who predominantly have hypertriglyceridemia. Increasing the statin dose to 80 mg (rosuvastatin to 40 mg) is associated with a threefold increase in liver toxicity or myopathy [34]. Therefore, treatment with non-statin cholesterol lowering agents, for example bile acid resin [35], niacin [36], fibrate [37] or cholesterol absorption inhibitor [38], is recommended for these patients.

Bile acid binding resins are non-absorbable anion exchange resins that bind bile acids in the intestinal lumen, preventing their absorption from the ilium and therefore increasing their fecal excretion. The liver responds by up-regulating cholesterol 7-alpha hydroxylase, which increases the conversion of cholesterol to bile acids, thereby reducing the cholesterol concentration in the hepatocyte [39]. Gastrointestinal disturbances, and drug and/or fat-soluble vitamin malabsorption, which were associated with early generation bile acid resins, have been overcome with new generation agents [35]. Bile acid resins can lower LDL-C approximately 10% to 25% which is appropriate for patients who need only moderate LDL-C lowering [35].

Niacin, or nicotinic acid, is the oldest lipid-lowering drug dating back to the 1950s [39]. Depending on dose and formulation, LDL-C reductions of 12% to 20% maybe anticipated, along with good reductions in triglycerides and 17% to 31% increase in high-density lipoprotein cholesterol (HDL-C). The major drawback to niacin use is its side effects, which include itching, headaches and hepatotoxicity. It is contraindicated in patients with active liver disease or unexplained abnormalities in liver function tests [39].

A cholesterol absorption inhibitor, more commonly known as Ezetimibe, impairs dietary and biliary cholesterol absorption at the brush border of the intestine without affecting the absorption of triglycerides or fat-soluble vitamins [19]. It has been shown to be well tolerated and effective in lowering LDL-C when used as a monotherapy or when adding to statin therapy. Ezetimibe at a dose of 10 mg/day reduced LDL-C by approximately 17% with no adverse effect of myopathy or liver toxicity [40,41]. However, recently concerns have been raised in respect to an independent atherogenic property of this drug, which appears to counteract its cholesterol-reducing action [42].

For patients who do not respond to a maximum dose of a statin and those who develop side effects with higher doses, a combination therapy of statin with one of the above agent, rather than an increase in the statin to high doses, may be more effective in achieving LDL-C goals and improving CAD outcomes while remaining at an acceptable safety profile [43]. Adding a bile acid resin or niacin to the statin can reduce LDL-C by approximately 50%, depending on the choice of statin and dosage prescribed [44,45]. Co-administering 10 mg of ezetimibe with any dose of statin reduced LDL-C levels by an additional 25%, compared with the usual 6% attained by doubling the statin dose [46]. However, even after treatment with a combination therapy, the majority of homozygous and minority of heterozyogotes FH patients may still have extremely raised LDL-C serum levels [47] and their risk of CAD remains unacceptably high.

Surgical interventions involving a portocaval shunt or an ileal bypass have yielded transient lowering of plasma LDL in these patients [48]. The preferred treatment at present is an aggressive programme of plasma apheresis or LDL apheresis, a physical procedure in which LDL is selectively removed from the blood by passing plasma over columns that bind the LDL. A small number of angiographic regression studies have been conducted and each weekly or fortnightly treatment has been demonstrated to lower LDL-C levels by about 55% and to delay onset and progression of atherosclerosis [49-53].

The most significant but also most aggressive metabolic correction is orthotopic liver transplantation in homozygous patients [54-56]. However, the morbidity and mortality risks as well as scarcity of donated organs are serious limitations.

Several novel therapeutic approaches have also been developed recently to lower LDL-C, either as monotherapy or in combination with statins [57] including; squalene synthase inhibitors [58], microsomal triglyceride transfer protein inhibitors [59,60], siRNA for PCSK9 [61] or for apolipoprotein B-100 [62] silencing, antisense PCSK9 [63], and antisense apolipoprotein B-100 (more commonly known as Mipomersen sodium (ISIS 301012)) [64,65].

In August 2010, Genzyme Corp. and Isis Pharmaceuticals Inc. announced the completion of the four phase 3 clinical trials that are required in the initial United States and European of regulatory filings for mipomersen. Filings for therapeutic use in homozygous FH are expected in the first half of 2011 [66]. These double-blinded, placebo-controlled clinical trials have been conducted at several locations worldwide. They involve heterozygous FH patients [67], homozygous FH patients [68], and patients with severe hypercholesterolemia [69]. The latter are defined by LDL-C levels ≥200 mg/dL and baseline cardiovascular disease (CVD) or by LDL-C levels ≥300 mg/dL without CVD. The trials also include patients with high cardiovascular risk [70] and high cholesterol levels as defined by LDL-C levels ≥100 mg/dL who were already taking maximally tolerated lipid-lowering medications.

At the end of the study, these patients had an average LDL-C reduction of 36-37% with no serious adverse effects. The reductions observed in the study were in addition to those achieved with the patients' existing maximally tolerated statin regimens. The trial also met each of its three secondary endpoints with statistically significant reductions in apo-B, non-HDL-cholesterol and total cholesterol. All trials also demonstrated manageable safety and tolerability profile of mipomersen.

Although each of these novel therapies effectively lowers LDL-C, challenges remain for clinical development in the assessment of long-term safety.

## Liver directed gene therapy for FH

Patients who have undergone liver transplantation and have experienced substantial reductions in LDL-C levels provide indirect evidence that gene therapy targeted towards the liver could be effective for this disease. While LDLR is expressed by the majority of body cells, hepatic reconstitution of LDLR expression alone may be sufficient for metabolic correction [71,72]. The liver is an attractive organ for FH gene delivery because of its large mass, its ability to synthesise large amounts of proteins, its central position in metabolism and its good accessibility through the portal vein [72,73].

The homozygous form of FH would be an excellent candidate for gene therapy since the plasma lipid profile, total cholesterol, LDL-C, HDL-C and LDL/HDL ratio, can be measured providing a convenient clinical endpoint to evaluate the response to therapy [71,72]. In addition, a sensitive non-invasive method using a scintillation camera is available to determine the location, magnitude, and duration of LDLR transgene expression which could provide functional transgene expression in gene therapy trials of FH [74]. Moreover, animal models are available, which include the Watanabe heritable hyperlipidemic (WHHL) rabbit [75], and rhesus monkeys [76], the ApoE-knockout (ApoE-/-) mouse [35], and the LDLR-knockout (LDLR-/-) mouse models [77]. The WHHL rabbit demonstrates hypercholesterolaemia due to natural deletion of 12 nucleotides in the LDL-binding domain of the LDLRcDNA [78]. This causes a delay in the post-translational processing of the 120 kDa LDLR-precursor to the 160 kDa mature form, leading to premature degradation of the mature form in the cytoplasm and consequently hypercholesterolaemia (700-1200 mg/dl at 12 months of age) [79]. The WHHL rabbit, therefore, demonstrates metabolic and clinical abnormalities similar to those in patients with FH and may be a more authentic FH model than the LDLR-/- or ApoE-/- mouse models [80] where the raised plasma cholesterol levels (225 ± 27 mg/dl) are lower unless the animals are subjected to a high cholesterol diet. There are also some intrinsic differences in the lipoprotein metabolism of mice compared to humans and rabbits. For instance, the main lipoprotein in plasma of FH patients and the WHHL rabbit is LDL, but in ApoE-/- mice [81] it is the VLDL fraction with apoB-48, and HDL and LDL in LDLR-/- mice [77]. The activity levels of the plasma cholesterol-ester transfer protein (CETP), which facilitates the transport of cholesteryl esters and triglycerides between the lipoproteins, and hence plays a role in LDL particle remodelling, are high in WHHL rabbits, although murine models lack this activity [80,82]. Consequently, HDL levels in the plasma are low in WHHL rabbits but high in mice and rats. In contrast, the ApoB-editing enzyme is not expressed in the liver of rabbits [80], but murine models do have ApoB-editing activity in the liver [81]. Therefore, apoB-48-containing VLDL is secreted from the liver in mice [80,81]. Selective breeding of WHHL rabbits resulted in coronary atherosclerosis-prone WHHL rabbits manifesting with features of coronary and aortic atherosclerosis and myocardial infarction, in contrast murine models are usually resistant to the development of myocardial infarction and features of coronary and aortic atherosclerosis [80]. For the above-described differences, the WHHL rabbit is thought to be a more authentic FH model similar to human subject (reviewed in [80]).

## Methods of gene delivery

Gene transfer can be performed either *ex vivo*, involving isolation of autologous cells from the patient, their *in vitro *genetic modification and selection followed by reimplantation of the transduced cells, or it can be done *in vivo*, where the vector is delivered directly to the organ [83]. The advantage of the *ex vivo *approach is that the transduction/transfection conditions can be carefully controlled and optimised and individual clones with the most desirable characteristics can be isolated to eliminate unmodified cells or cells with deleterious mutations before re-implantation. While this approach is laborious and time consuming, it may also offer significantly greater safety and control with respect to vector mediated mutagenesis and possible germline transmission of the transferred genes, which is a risk of *in vivo *gene delivery. The disadvantages of the *ex vivo *approach are failure of cell engraftment and difficulties in returning the cells to the patient due to disease manifestations such as portal vein hypertension [83,84].

The *in vivo *approach eliminates the need for engraftment after re-implantation and is therefore easier to perform, more cost effective and may be more applicable for use in countries with limited laboratory resources. The gene transfer vector is injected into the bloodstream (systemic delivery) aiming at somatic cell delivery only or by use of specific cell targeting, preferentially to the tissues of interest (targeted delivery). Organ specific delivery of the gene transfer vector includes intrahepatic injection or selective intravasular application routes. Disadvantages of *in vivo *gene transfer are vector dilution, ectopic transgene expression and non-targeted, random, potentially genotoxic insertion into the host genome.

## Gene transfer systems

In addition to the method chosen for delivery, successful treatment of FH would ideally require safe and efficient gene transfer vectors that provide appropriate and sustained levels of transgene expression and long-term survival of treated cells. The use of a liver specific promoter would be the most physiological approach to achieve this. However, because of present problems in transfection-efficiency, strong heterologous promoters are commonly used instead for proof of principles studies on the effectiveness of lipid-lowering. The development of more effective vectors to achieve this remains a formidable challenge to gene therapy. The properties required of such a vector system and those that should be avoided are listed in Table 1.

**Table 1:** The properties required for development of an ideal vector system and those that need to be avoided.

Properties needed	Properties to be avoided
Stable high titre vectors	Vector degradation

Simple and reproducible production	Replication competent virus

Unlimited packaging capacity	Expression of undesirable viral proteins

Efficient gene transfer to the target cells	Germline gene transmission

Controlled genomic integration	Insertional mutagenesis

Regulated normal levels of expression	Inappropriate toxic expression

Ability to repeat delivery if needed	Severe immune response against vector system

Long-term expression	Immune response against transgene products

Gene transfer vectors are generally classified under two categories; they are either non-viral or virus mediated gene transfer systems.

## Non-viral gene transfer systems

Gene therapy vectors based on modified viruses are unquestionably the most effective gene delivery systems in use today. Their efficacy at gene transfer is however tempered by their potential toxicity [85,86]. An ideal vector for human gene therapy should deliver sustainable therapeutic levels of gene expression without compromising the viability of the host (at either the cellular or somatic level) in any way. Non-viral vectors are attractive alternatives to viral gene delivery systems because of their low toxicity, relatively easy production and great versatility [87].

Most of the non-viral vectors that have been described for gene therapy are based on complimentary DNA (cDNA) gene sequences driven by highly active promoters. The DNA in these vectors is typically formulated with cationic agents to form complexes, which protect the DNA and facilitate cell entry [87,88]. DNA can, however, be driven into cells by physical means and the liver is particularly amenable to gene delivery via hydrodynamic delivery. Mahato *et al *reported that a standard tail vein injection of naked DNA into mice resulted in almost no gene expression in major organs due to its rapid *in vivo *degradation by nucleases and clearance by the monocular phagocyte system [89]. However, a very rapid injection of a large volume of naked plasmid DNA solution (e.g. 5-10 μg of DNA in 2.5 ml saline, which is almost equivalent to the blood volume of the animal, within 5-7 seconds) *via *the same route induced efficient gene transfer particularly in the liver [90]. This procedure was applied in one of the first non-viral approaches to reverse hypercholesterolaemia in an FH model. In these experiments a DNA construct was produced which encoded a fusion-protein consisting of a soluble form of the LDLR combined with transferrin. The strategy of this approach was based on the ability of the fusion protein to be capable of binding both plasma LDL and the cellular transferrin receptor. When applied *in vivo *following hydrodynamic injection [91], this protein was shown to bind circulating plasma LDL and to mediate its clearance through the transferrin receptor on hepatocytes. Although the system proved functional, a statistically significant change in the lipoprotein profile of an animal model was not demonstrated and the possible immunogenicity of the fusion protein potentially precludes its utility.

In contrast to using a cDNA expression cassette, the use of a complete genomic DNA locus to deliver an intact transgene with its native promoter, exons, all intervening introns, and regulatory regions with flanking non-coding genomic DNA sequences may allow regulated complementation of LDLR deficiency in the liver of hypercholesterolaemic animals. In 2003, a bacterial artificial chromosome containing the entire LDLR genomic locus and based on the Epstein Barr Virus (EBV)-retention system was delivered to LDLR deficient Chinese hamster ovary cell line (CHOldlA7) [92], and achieved correction of the cells' deficiency phenotype [93]. This vector construct was able to mediate LDLR expression at significant levels in the CHOldlA7 cells and in human fibroblasts derived from FH patients for 3 months and to retain the classical expression regulation by sterol levels in these cells. These initial studies paved the way for the development a more sophisticated vector which utilised a Scaffold Matrix Attachment Region (S/MAR) rather than a potentially toxic viral component to provide episomal maintenance [94]. In this study the LDLR genomic locus was incorporated into an HSV-1 amplicon vector, which was shown to remain episomal for 11 weeks and provided the complete restoration of human low density lipoprotein receptor LDLR function in CHOldlA7 cells to physiological levels. The vector comprised the LDLR gene driven by 10 kb of the human LDLR genomic promoter region including the elements, which are essential for physiologically regulated expression. By utilizing the genomic promoter region it was demonstrated that long-term, physiologically regulated gene expression and complementation of receptor deficiency could be obtained in culture for at least 240 cell-generations. Importantly, this vector was shown to be sensitive to the presence of sterols or statins, which modify the activity of the LDLR promoter. These *in vitro *studies finally lead to the successful administration of genomic LDLR vectors *in vivo *via hydrodynamic delivery [95,96]. When administered hydrodynamically in mice it was demonstrated that efficient liver-specific delivery and statin-sensitive expression could be obtained for up to 9 months following delivery [96].

While the majority of studies were focused on treating FH by inhibition of hypercholesterolaemia through up-regulation of LDLR or other surrogate lipoprotein receptors (as will be discussed later), an alternative approach was to down-regulate apoB-100 LDLR-ligand or PCSK9 [63] expression. Down-regulation of apoB-100 by continues intravenous/subcutaneous administration of Mipomersen antisense apolipoprotein B-100 oligonucleotide had been attempted in several clinical trials achieving average LDL-C reduction of 36-37%. Although, the most common adverse event of these trials was erythema at the injection site due to the protocol [64,65,97-102], challenges remain for clinical development in the assessment of long-term safety.

## Recombinant virus-based gene transfer systems

Recombinant viral vectors are usually more effective than non-viral vectors in mediating cell entry and nuclear transfer of therapeutic genes in the target cells. In addition natural tropism of viral envelopes and serotypes can be employed to achieve targeting selectivity for particular host cells. Most of these vectors have mechanisms to avoid intracellular degradation and overcome cellular and immunological barriers to the delivery of the genetic cargo. Generation of a virus vector requires the transformation of a potentially harmful virus from a pathogen into a gene transfer agent whilst retaining the viral infectivity. Hence, the first step is to make the vector replication defective (incapable of producing infectious viral particles in the host's target cells). Replication deficient viral vectors are developed by deletion of crucial genes in the virus genome, which are then generally replaced by the therapeutic gene. The elements removed in this way have to be provided *in trans *in order to support vector production. This can be achieved by use of a helper virus or a packaging (production) cell line transfected with the plasmids expressing the genes coding for the required structural virus components and replication proteins. Helper virus must be purified away from the final vector batches intended for safe gene delivery.

Recombinant viral vectors presently used are generally classified under two categories; integrating or non-integrating viral vectors [103]. This distinction is an important determinant of the suitability of each vector for a particular application. Present integrating vectors rely on random insertion of the transgenic DNA into the cell's genome, leading to stable integration and subsequent passage to the cell's progeny. This gene insertion via non-homologous end joining of vector DNA to that of the host using a virus integrase can be most efficiently achieved using retroviral or lenti-retroviral vectors. Adeno-associated viral vector integration is less frequent than that of the unmodified parent virus, which targets preferentially into chromosome 19 and does not show the locus specificity of wild-type virus. It is important to note that integration does not guarantee stable transgene expression due to host mediated gradual silencing of gene expression over time [104], immuno-elimination or physiological cell death of gene modified cells.

Non-integrative vectors such as adenovirus and herpes simplex viral vectors allow transient episomal expression of a foreign gene in the target cells. Because of their episomal maintenance, the transferred genes are usually lost over time by dilution at cell division in actively dividing cells or by degradation in non-dividing cells [105]. A non-integrative vector could ideally be delivered repeatedly if required, as long as no immunological reactions develop against the vector or transgenic protein. Unlike adenovirus and herpes simplex viruses, EBV is stably maintained without integration in permissive proliferating cells due to the EBV nuclear antigen 1 protein-mediated replication and segregation, providing long-term transgene expression [106]. It is however unlikely to be used in a clinical setting due to its association with Burkitt's lymphoma. The properties of the most commonly used viral vectors are summarised in Table 2.

**Table 2:** General properties of the most commonly used viral vectors.

Properties	Adenoviruses	AAV	Retroviruses	Lentiviruses
**Wild type viruses**	36 kb ds linear DNA	4.7 kb ssDNA	9.2 kb Diploid +ssRNA	8-10 kb Diploid +ssRNA

**Pre-existing host antibodies**	Yes	Yes	Unlikely	Unlikely but (may be in HIV +ve individuals)

**Packaging capacity**	8-30 kb	4 kb	<8 kb	8 kb expected

**Viral titre (particles/ml)**	>10^13^	>10^12^	>10^9^	10^9^

**Stability**	Good	Good	Good	Not tested

**Integration**	No	<10% integrated	Yes	Yes

**Cellular localisation**	Nuclear	Nuclear	Nuclear	Nuclear

**Cell range**	Non-replicating and replicating	Non-replicating and replicating	Replicating only	Non-replicating and replicating

**Levels of expression**	Very high	Moderate	Moderate	Moderate

**Duration of expression**	Transient	Long	Long, but subject to shutdown	Long

**Immune response**	Extensive	Not known	Few neutralis-ing antibodies	Not known

**Safety issues**	Inflammatory and toxicity	Rearrangement and inflammatory	Insertional mutagenesis	Insertional mutagenesis

**Main advantages**	Extremely efficient transduction of most tissues	Non-inflammatory Non-pathogenic	Long-term gene transfer in dividing cells	Long-term gene transfer in dividing cells

**Main disadvantages**	Capsid mediates a potent inflammatory response	Small packaging capacity	Transduces only dividing cells and potential for oncogenesis	Potential for oncogenesis

kb, kilo base; ssDNA, single stranded DNA; dsDNA, double stranded DNA.

### Retrovirus based vectors

**Figure 2:**
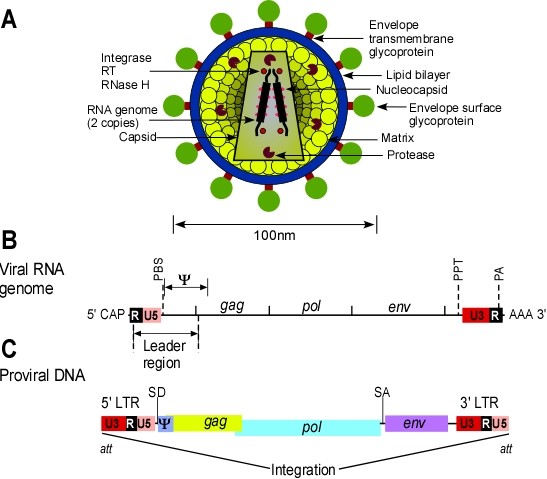
**Retrovirus virion, viral genome and RNA transcript**. **A.) **Schematic diagram of virion structure. **B.) **The genome organisation of an oncoretrovirus provirus DNA; locations of all genes and the LTR are indicated. **C.) **Viral RNA transcripts. The full-length transcript serves as the RNA genome and as a messenger RNA for Gag and Gag/Pol polyproteins. The Env is translated from the spliced transcript.

Retroviruses (RVs) are a large family of enveloped RNA viruses, which are generally classified into three subfamilies; oncoretroviruses, lentiviruses and spumaviruses (foamy viruses). The RV particle is composed of two copies of an RNA genome held together primarily by a sequence called the dimer linkage, which is termed the leader region and found in some cases in *gag *coding region. The genome is surrounded by a spherical or cylindrical shaped core and an enveloped glycoprotein, (Figure 2-A). Upon infection, RVs are able to convert their RNA genome in the host cytoplasm to DNA through reverse transcription (RT) [107].

The genome size of simple RVs is approximately 8-12 kb and comprises three main genes; the group specific antigen encoding gene (*gag*), the polymerase encoding gene (*pol*), and the envelope glycoprotein encoding gene (*env*), which are flanked by elements called long terminal repeats (*LTR*s), Figure 2-B. The *gag *gene encodes the viral structural core proteins, which form the matrix, capsid and nucleocapsid, generated by protease cleavage of the Gag precursor protein. The *pol *gene expresses a complex of enzymes that are involved in particle maturation (protease), DNA metabolism (reverse transcriptase) and proviral integration (integrase). These enzymes are usually derived from the Gag/Pol precursor. The *env *gene encodes the surface glycoprotein and the transmembrane protein of the virion, which form a complex that interacts specifically with cellular receptor proteins. The genes in the viral DNA are bracketed by the *LTR*s, which define the beginning and the end of the viral genome. The *LTR*s are identical sequences that can be divided into three elements. *U3 *is derived from a sequence unique to the 3' end of the RNA, *R *is derived from a sequence repeated at both ends of the RNA, and *U5 *is derived from a sequence unique to the 5' end of the RNA. The genesis of the *LTR *elements lies in the process of reverse transcription. *U3 *contains most of the transcriptional control elements of the provirus (viral genome, which has integrated into the chromosomal DNA of a cell), which include the promoter and multiple enhancer sequences responsive to cellular and in some cases viral transcriptional activator proteins. The site of transcription initiation is at the boundary between *U3 *and *R *of the 5' *LTR *and the site of poly(A) addition is at the boundary between *R *and *U5 *at the 3' *LTR*, as shown in Figure 2-C. The other boundaries of *U3 *and *U5 *are determined by the primer binding site (*PBS*) and the polypurine tract (*PTT*), which are important for reverse transcription. Just downstream of the 3' end of the 5' *LTR*, is a short packaging sequence (Psi,Ψ), which extends into *gag *and is responsible for encapsidation of the two viral RNA genomes into the capsid. The *att *sequences at the ends of the 5' and 3' *LTR*s are necessary for proviral integration [107].

The life cycle of a RV starts with high affinity binding of the viral envelope glycoprotein to its receptor on the outer layer of the cell membrane. This interaction leads to the fusion of the lipid envelope surrounding the virus, with the target cell membrane. Cell entry of the viral capsid containing the RNA genome allows the reverse transcriptase enzyme to copy the viral RNA genome into a double-stranded DNA, which becomes associated with viral proteins to form what is called a pre-integration complex (PIC). The PIC translocates to the nucleus where the viral enzyme integrase, which is part of PIC, mediates integration of the provirus DNA sequence into the chromosomal DNA of the host cell. The inserted sequence (provirus) is flanked by complete copies of *LTR *sequences. The 5' *LTR *drives transcription of the RV genome, which gives rise to RNA that codes for the viral proteins Gag, Pol and Env as well as for the viral RNA genome, Figure 2-C. Gag and Gag/Pol proteins assemble as viral core particles at the plasma membrane which package the viral RNA genomes and bud from the cell membrane enveloped with plasma membrane lipid from the host, in which virus derived Env glycoproteins are embedded [107].

**Figure 3:**
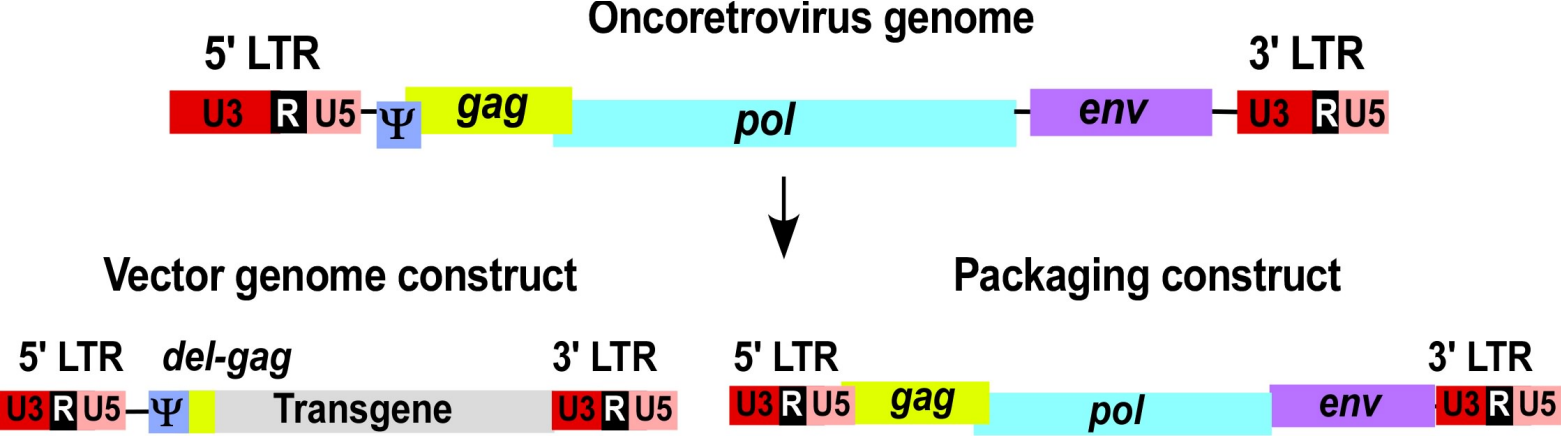
**The genomic organisation of MoMLV and the basic retroviral vector design**.

The first generation replication defective retroviral vectors were developed using Molony Murine Leukaemia Virus (MoMLV) as a prototype. Replication defective vector particles were produced by a deconstruction strategy, which aims to dissect/segregate the viral genome into two transcriptional units (plasmid constructs). They are the vector genome and the packaging constructs, Figure 3. The vector genome retains all the necessary *cis *elements of the vectors and is generated by replacing viral protein encoding sequences later to be provided in trans with the transgene of interest. Both the Ψ signal that is essential for packaging of the vector genome into the capsid and the viral *LTR*s, which are necessary for proviral integration, remain in the vector genome construct. Expression of the transgenic protein is driven by the promoter in the *U3 *region of the 5' *LTR*. The packaging construct provides all of the viral proteins in *trans *to the vector genome construct (Gag, Pol and Env). The packaging signal is deleted from the packaging construct to prevent its incorporation into viral particles. When both the vector genome and packaging constructs are present in a producer cell, retroviral vector particles, which are capable of delivering the vector genome with its inserted gene into new target cells, are released [108]. The process of gene transfer by such a vector is referred to as transduction. When these two constructs are present in cells in an integrated form, the cell becomes a stable virus producer. Alternatively, virus can also be produced for a short period of time after transient co-transfection of the viral genome alongside with packaging plasmids.

The basic arrangement described above is functional but unsatisfactory for several reasons. Firstly, the sequence overlap that remains between the vector and packaging constructs could result in recombination to form infectious replication competent retrovirus (RCR). The overlap exists principally because extensive sequences of the *gag *gene are retained in the vector construct to enhance the efficiency of packaging, although Gag protein production is prevented by mutation. In addition, overlapping sequences also exist because the *LTR*s are retained in the packaging construct to provide both promoter and poly-adenylation sequences. Secondly, the early MoMLV based vectors were established in murine NIH 3T3 packaging cell lines, therefore, the possibility for RCR generation through recombination between vector constructs and defective endogenous MoMLV-like sequences present in the target cells cannot be excluded [109]. Thirdly, vector particles produced in murine cells can be sensitive to host compliment mediated inactivation after *in vivo *gene delivery [110,111].

**Figure 4:**
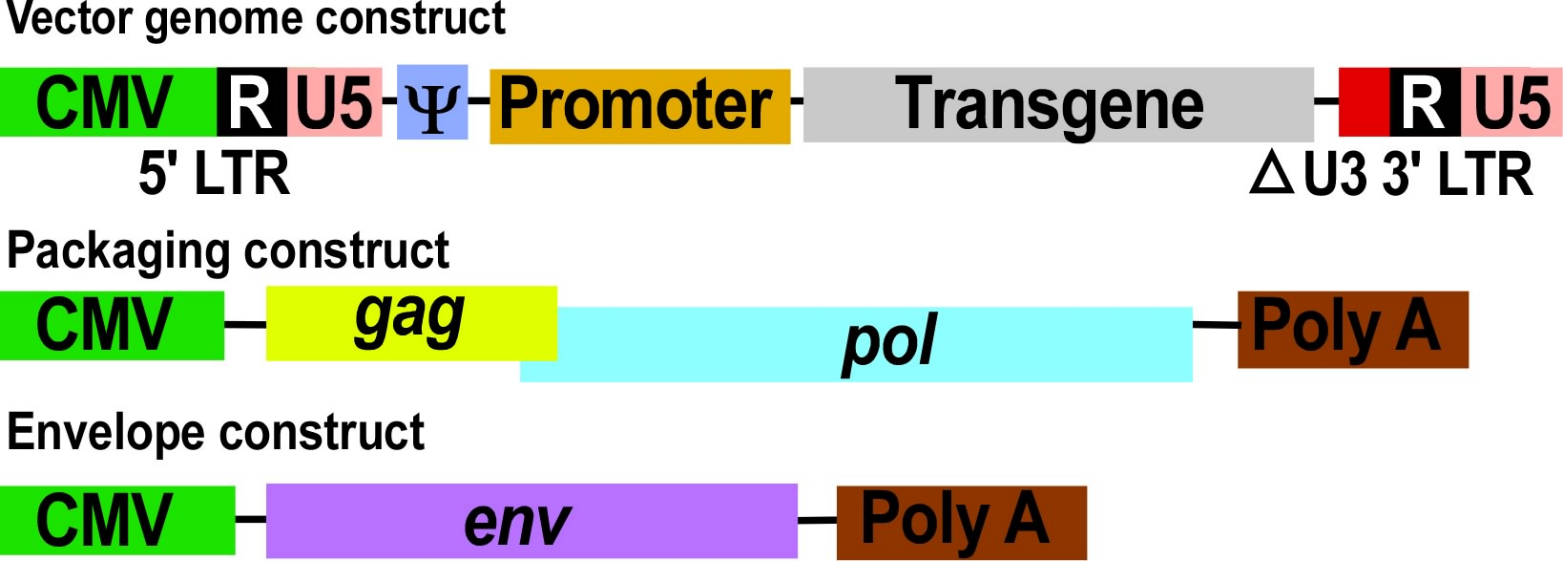
**Schematic representation of the improved retrovirus three plasmid co-transfection system**. Viral genome is segregated into three expression plasmids; the vector genome, the Gag/Pol expressing plasmid, and the envelope expressing plasmid. For generation of viral particles, these plasmids are co-transfected into the HEK 293 producer cells, and virus is released into the supernatant.

In order to minimise the risk of RCR production, an improved vector system was designed by segregating the *gag*/*pol *and *env *genes present on the packaging construct, onto discrete expression units, Figure 4. The risk of recombination was also further reduced by the use of heterologous envelope proteins that are derived from alternative viruses with no homology to parental virus sequences but are still able to be incorporated into the viral particle (a process referred as pseudotyping). Pseudotyping may also alter the tropism of the viral vector and can be used as a powerful tool for cell targeting different host tissues. Pseudotyping of MoMLV and other RVs with the murine ecotropic (recognising only receptors present on mouse cells), amphotropic (interacting with receptors on both mouse and human cells) or the vesicular stomatitis virus glycoprotein (VSV-G) envelopes (with broad host range including mammalian and even insect cells) has been achieved and proven useful [112,113]. In this improved virus production system, part of the *gag *sequence present on the first generation MoMLV vector is removed from the vector genome without significant loss of packaging efficiency (it was subsequently found that part of the gag region is essential for efficient vector packaging) [114], Figure 4.

The problem of overlapping sequences between the vector and the packaging construct has been solved by using heterologous promoters and polyadenylation signals to drive structural gene expression from the packaging constructs. Strong heterologous promoters like cytomegalovirus (CMV) can provide high virus titre production circumventing the limited titre offered by he MoMLV *LTR*s that give low-level gene expression in producer cell lines not of murine origin. In the vector genome construct itself, heterologous promoters have been used to replace the 5' *U3 *promoter. In addition, the 3' *U3 *sequences can be significantly deleted as long as the sequences necessary for recognition by the integrase protein are retained. This is the basis of self inactivating (SIN) vectors where deletion of the viral promoter and enhancer regions in the 3' *U3 *are duplicated during reverse transcription in the 5' LTR to prevent *LTR*-driven transcription in infected host cells which could result in the expression of downstream inserted proto-oncogenes [115]. Transgene expression in these vectors is therefore typically and exclusively driven by an internal heterologous promoter, which allows the use of regulated and/or tissue specific expression. Finally, a non-murine producer cell line was used for vector production to prevent the possible generation of RCR through recombination with endogenous MoMLV-like sequences [110].

In the latest generation of RV based-vectors, improvements have also been made in the vector titre (number of colony-forming units per ml) by the development of transient plasmid co-transfection systems, which are capable of producing very high vector titres for a short period of time in the highly transfectable HEK 293 (human embryonic kidney epithelial cells) cell line [110]. Also some human cells used to generate packaging cell lines can produce a complement-resistant retroviral vector [111]. Transfection of HEK 293T cells using SV40 large T antigen to improve vector load and hence vector titre are used also to circumvent the cytotoxicity of the highly desirable VSV-G envelope that provides broad host range infection.

Recombinant MoMLV-based vectors produced by the strategy described above are efficient gene transfer vehicles, reaching transfer levels *in vitro *of close to 100%. They can be produced at a high titre (10^9 ^infectious units (lU)/ml) and have the capacity to infect a wide variety of dividing cells including hepatocytes. The RV vector genome can also provide transfer of RNA of approximately 7.5 kb in length.

The critical limitation to the use of RVs is their inability to infect non-dividing cells and as the liver is an only slowly proliferating tissue these vectors are not ideal for LDLR gene delivery to hepatocytes. Therefore, for direct *in vivo *transduction of the liver, cells have to be either in a naturally dividing state or to be induced to divide. Alternatively, the vectors can be used for *ex vivo *treatment.

Hypercholesterolaemia has been ameliorated by RV-based vectors using *ex vivo *gene delivery in numerous experimental studies. The original procedure used for liver-directed gene therapy of FH was based on the *ex vivo *approach, which involved re-infusion of autologous hepatocytes that had been removed from a WHHL rabbit and subjected to *in vitro *genetic correction with RV vectors based on MoMLV. Animals transplanted with LDLR transduced celIs demonstrated a 30-50% reduction in total serum cholesterol levels persistent for the duration of the experiment (122 days). Recombinant derived LDLR mRNA was detected in liver cells for 6 months. There was no apparent immunological response to the recombinant derived rabbit LDLR [116]. This study illustrated the potential of the *ex vivo *approach to ameliorate hyperlipidaemia associated with FH using a RV-based vector.

In preparation for human trials with RV-based vectors, the efficacy, safety and feasibility of *ex vivo *gene therapy for FH was further documented in non-human primates [117,118]. Three baboons were subjected to a partial hepatectomy and their hepatocytes were isolated, cultured, and transduced with a RV containing the human low-density lipoprotein (hLDLRcDNA) sequence. Infusion of the genetically modified hepatocytes was performed through a catheter that had been placed into the inferior mesenteric vein at the time of liver resection. The baboons tolerated the procedures and were monitored for up to eight months [117]. The safety and efficacy of the *ex vivo *approach for delivery of gene transduced hepatocytes via the mesenteric circulation was further documented in a canine model [118].

The above studies demonstrated the feasibility and safety of the *ex vivo *approach, which was then carried out on a human patient in the first clinical trial for FH published in 1994. In this trial a 29 year-old woman with a homozygous receptor defective FH was subjected to *ex vivo *gene therapy using an amphotropic RV-based vector expressing human LDLRcDNA under control of the CMV enhanced chicken β-actin promoter. The patient tolerated the procedure and *in situ *hybridisation of liver tissue four months after therapy revealed evidence for engraftment of transgene expressing cells. The patient's LDL/HDL ratio declined from 10-13 before vector delivery to 5-8 after vector delivery, an improvement that remained stable for the duration of the reported observation (18 months). However, kinetic studies of LDL metabolism including LDL binding, uptake and degradation were not presented [119]. This trial was severely criticised with respect to both the suitability of the patient for this therapeutic intervention and for the aggressiveness of the protocol, which involved a 25% hepatectomy [120].

Grossman et al then reported four additional homozygous FH patients subjected to a surgical resection of the left lateral segment of the liver and re-infusion of the genetically modified hepatocytes [121]. The patients tolerated the infusions of autologous hepatocytes well without complications. Liver biopsies performed four months after treatment revealed LDLR transgene expression in a limited number of hepatocytes by *in situ *hybridisation in all four subjects. One of four patients had a significant and prolonged reduction of about 20% in his LDL-C levels. Kinetic studies of the LDL metabolism demonstrated that LDL catabolism was increased in the same patient, which was consistent with increased LDLR expression [121]. The reason for the only marginally successful lowering of cholesterol levels and the variable metabolic responses observed in the five subjects studied are presumably due to low gene transfer efficiency or low expression levels [121]. The variable metabolic response observed following low-level genetic reconstitution in the five patients precluded a broader application of *ex vivo *liver-directed gene therapy with RV based vectors, pending improvement of vector efficiency. The following sections review the preclinical work towards this goal with alternative vector system.

### Adenovirus based vectors

**Figure 5:**
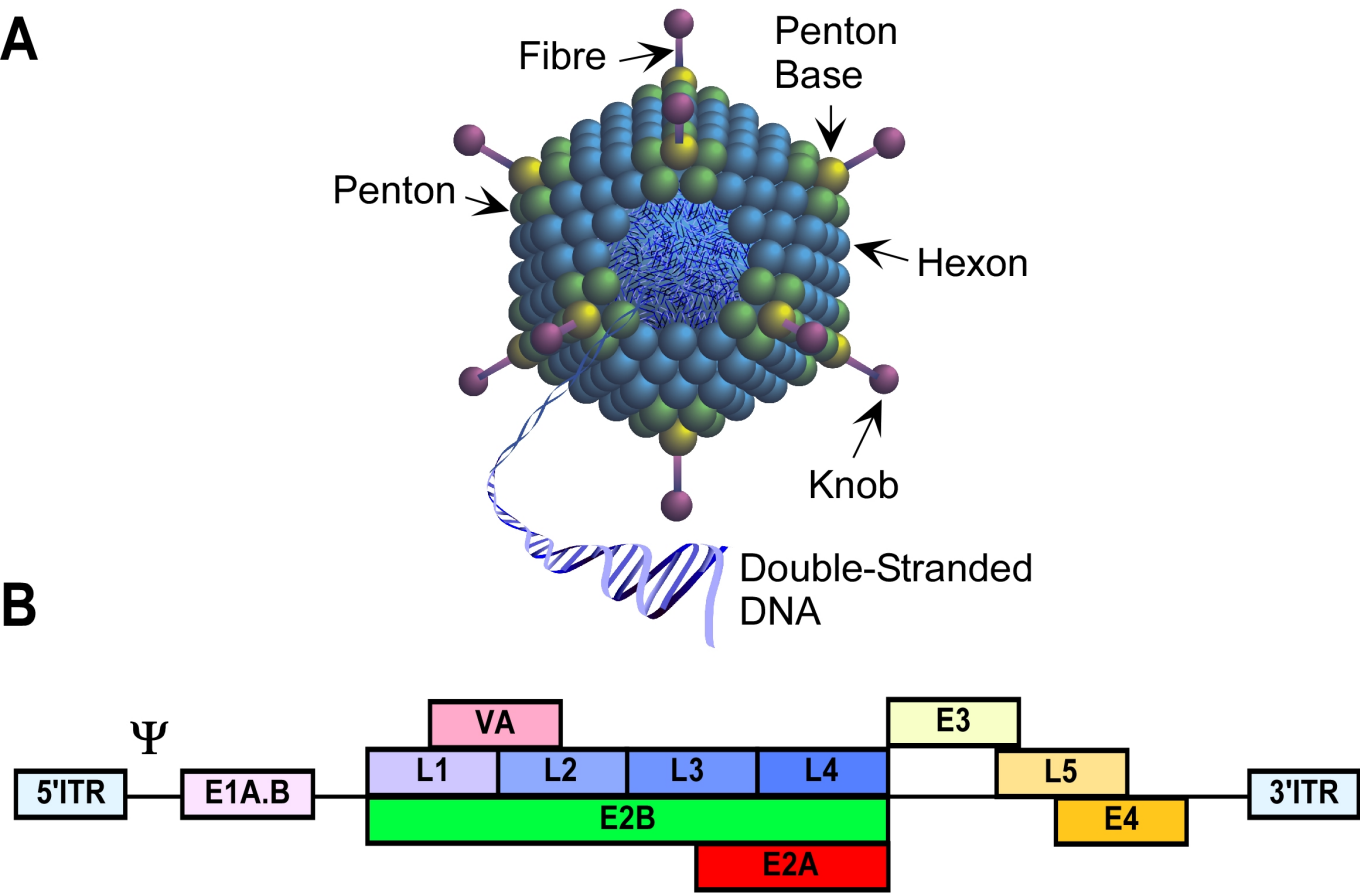
**Features of adenovirus particle**. **A.) **Structural features of Ad. **B.) **Genomic organisation of wild type Ad. Reproduced with modifications from original diagram provided kindly by Dr Simon Waddington.

Adenoviruses (Ads) are icosahedral particles consisting of linear, double stranded DNA with a non-enveloped virion, (Figure 5-A). There are at least 50 different human adenovirus serotypes with an approximate genome size of 36 kb. The Ad genome (Figure 5-B) is intimately associated with viral proteins (core) and is packaged in the viral capsid, which consists primarily of three proteins; hexon, penton base and fibre-knob. After infection, the virus genome does not integrate into the host chromosomal DNA, instead it is replicated as an episomal (extra-chromosomal) element in the host nucleus [122].

Because of the ability of adenoviral vectors to infect a broad range of mammalian cell types regardless of their replication status, they have been widely used for a variety of gene transfer applications *in vitro *[123], *in vivo *[124] and in clinical trials [125]. Most adenoviral vectors currently used are derived from serotypes 2 or 5, which are endemic and cause upper respiratory tract infection in humans. Most human individuals have become immune-sensitised by natural infection during childhood [83].

Vectors derived from serotypes 2 and 5 enter the cells after attachment to the cellular receptor CAR (coxsackievirus and adenovirus receptor), through the knob of the fiber [126]. Virus entry occurs then through cIathrin-mediated endocytosis after binding of the penton base to integrins [127]. It is noteworthy that differences in the tropism of various Ad serotypes indicate that besides CAR, other cellular receptors also contribute, suggesting that the host range of Ad vectors can be altered by use of alternative serotypes.

The first generation of replication deficient Ad vectors was constructed by replacing one or two viral early (E1 and E2) genes, which are essential for viral replication, with the transcriptional cassette of interest containing an enhancer-promoter element and the desired gene. Vectors in such a configuration have a packaging capacity of 6.5-8.3 kb. The recombinant vectors are replicated in cells that express the products of the E1 and/or E2 genes. Purified high titre stocks of 10^11^-10^12 ^Ad particles per ml, can be generated and allow high efficiency Ad mediated gene transfer with strong tropism for the liver. Cells that were transduced with these vectors express adenoviral genes at low levels, in addition to the transgenic protein [128].

The utility of replication defective first-generation recombinant Ad to mediate hLDLR gene transfer in hepatocytes derived from FH patients was first examined and documented in 1993 [123], using the β-actin promoter. The level of recombinant-derived LDLR protein in transduced FH hepatocytes exceeded the endogenous levels by at least 20-folds.

Reversal of hypercholesterolaemia was then demonstrated in LDLR-/- mice fed with a high cholesterol diet after intravenous injection of a replication-defective Ad encoding the hLDLR driven by CMV promoter. This *in vivo *approach resulted in reduction of the elevated intermediate density lipoprotein (IDL)/LDL ratio to normal levels, four days after vector delivery [129]. Similarly, injection of a replication-defective Ad encoding the hLDLR driven by an optimised CMV promoter into the portal vein of WHHL rabbits, resulted in over-expression of hLDLR in the majority of hepatocytes that exceeded the levels in normal human liver by at least 10 fold. Transgene expression was stable for 7-10 days but diminished to undetectable levels within three weeks [130]. Similar studies were also conducted on WHHL rabbits with Ad vectors containing rabbit LDLRcDNA [131] or human LDLRcDNA [132]. These studies also resulted in strong but transient transgene expression. However, the high level of LDLR expression and substantial reduction of total and LDL cholesterol achieved by adenovirus LDLR gene transfer in these animal models led to a massive intracellular lipid (cholesterol and cholesterol ester) deposition in transduced cells [130,133]. This accumulation resulted from non-physiological over-expression of LDLR mediated by the Ad vector, causing pathological intracellular accumulation of the lipid that could not be compensated by the hepatic cell metabolism [133,134].

The transient expression was not solely due to the episomal nature of Ad infection but also a result of host immune responses against adenoviral proteins [124,135,136]. Co-administration of an Ad vector encoding hLDLR driven by a CMV promoter, with a blocking antibody directed against CD154 (CD40 ligand) to suppress immune responses against the vector and foreign transgene product in LDLR -/- mice, resulted in long-term expression of LDLR and maintained cholesterol levels within and below the normal range for at least 92 days post vector delivery. The loss of hLDLR expression in non anti-CD154-treated mice also demonstrated the importance of the host immune response against vector and transgene products [137].

In direct response to these immunological reactions and vector cytotoxicity, helper dependent adenovirus (HD-Ad) vectors were developed, in which additional viral coding sequences were deleted [138]. This also increases the insert capacity of the vector to approximately 30 kb. Nomura and colleagues [139] compared the efficiency of monkey LDLR gene therapy with that of monkey very low density lipoprotein receptor (VLDLR) gene therapy, using HD-Ad. High cholesterol diet fed LDLR-/- mice were injected with a single intravenous application of high (1.5 × 10^13 ^vector particles (vp)/kg) and low (5 × 10^12 ^vp/kg) doses of HD-Ad. Throughout the 24-week experiment, plasma cholesterol of LDLR-treated mice was lower than that of VLDLR-treated mice. Anti-LDLR antibodies developed in 2 of 10 mice treated with high-dose HD-Ad-LDLR but in none (0/14) of the other treatment groups. The antibody titre in the high-dose experiments was significantly above background, but was three orders of magnitude lower than that seen following first generation Ad-LDLR treatment, indicating that the marked pro-inflammatory adenoviral protein expression following FG-Ad-LDLR gene transfer could have acted as an adjuvant that stimulated antibody production in these mice. Long-term efficacy of low-dose HD-Ad-LDLR injected into 12-week old LDLR-/- mice was tested and after 60 weeks, atherosclerosis lesions covered approximately 50% of the surface of aortas of control mice whereas aortas of treated mice were essentially lesion-free. The lipid lowering effect of HD-Ad-LDLR lasted at least 108 weeks (>2 years) when all control mice had died [139].

Despite the reported improvements achieved by HD-Ad, the cytotoxic effect resulting from immune response to high titre (3.8 × 10^13 ^lU/mI) administration of a 2^nd ^generation adenoviral vector, which led to the unfortunate death of a patient in a non-FH clinical trial [125] stopped any further *in vivo *adenoviral vector delivery trials, pending improvement in vector design. In an attempt to address this issue, Jacobs and colleagues investigated the use of a relatively low dose (5 × 10^10 ^particles) of second generation E1E3E4-deleted adenoviral vectors for transfer of the LDLR or VLDLR, under control of the hepatocyte-specific human *α*_*1*_*-antitrypsin *promoter and 4 copies of the human *apo E *enhancer, into C57BL/6 LDLR-/- mice [140]. Evaluation was performed for 30 weeks after vector delivery in male and female mice fed either standard chow or an atherogenic diet. Compared to control mice, AdLDLR and AdVLDLR persistently decreased plasma non-HDL cholesterol in both sexes and on both diets and potently inhibit development of atherosclerosis in the ascending aorta. The non-physiologically regulated over-expression of LDLR or VLDLR, transferred by E1E3E4-deleted adenoviral vectors, significantly reduces tissue cholesterol levels in myocardium, quadriceps muscle, and kidney and does not lead to pathological intracellular accumulation of cholesterol and cholesterol esters in hepatocytes. The effectiveness of the vectors and expression cassette used in this study is stressed by the fact that, using vector doses that are 2-7.5-fold lower compared to those in other studies [139,141], equivalent results were obtained in terms of lipid lowering and reduction of atherosclerosis [140]. However, immune response to the vector system to evaluate potential development of neutralizing antibody or immune rejection to the transgene and/or vector has not been shown.

Adenoviral based vectors still remain the most efficient class of vector in terms of delivering to and expressing their genetic cargo in the cells of most tissues. However, because of their transient expression characteristics, while they remain useful for proof of principle for gene therapy they are not the vector of choice for the treatment of inherited monogenic diseases but will probably find application in the treatment of cancer in which cellular toxicity and immunogenicity might even enhance their anti-tumour effects [142].

### Adeno-associated virus vectors

**Figure 6:**
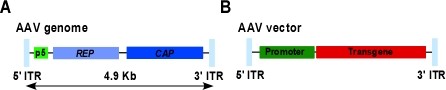
**Genomic organisation of the AAV and basic AAV vector design**.

Vectors based on adeno-associated virus (AAV), a small (20-25 nm) non-enveloped DNA virus (Figure 6) that is non-pathogenic and replication-defective, have a number of attributes that make them suitable for gene transfer to the liver for the treatment of FH. A single administration of recombinant AAV (rAAV) into the liver results in long-term transgenic protein expression without toxicity in a variety of animal models [143]. These pre-clinical studies have lead to phase I/II trials of liver gene transfer for diseases such as haemophilia [144] for example, using AAV serotype 2, the first isolate to be characterised.

There are several current obstacles to AAV gene therapy that need to be addressed. Although AAV is not known to cause human disease, 85% of the adult population is sero-positive for AAV capsid proteins [145] and wild type (wt) AAV2 is endemic to humans. Thus most of the patients that participated in clinical trials are likely to have had pre-existing immunity to the serotype employed, as a result of prior natural infection. Cytotoxic T-cells resulting from wt-AAV infection can eliminate transduced cells and anti-AAV2 antibodies are able to block or reduce gene transfer with rAAV2 vectors. These factors may have limited transgenic hFIX protein expression in a recent hemophilia B gene therapy trial [144]. Switching the capsid protein to other AAV serotypes that are less prevalent in humans can overcome these immunological problems [146]. There are several AAV serotypes available that may prove useful in the future for clinical translation.

Currently a high multiplicity of infection is needed to achieve therapeutic AAV mediated gene transfer. Efficient transduction of target cells is blocked at several levels during AAV cell infection and movement of the vector into the nucleus. The host gender appears to be an important consideration, since in mice exogenous androgens can increase stable hepatocyte gene transfer in females to levels observed in male mice [147]. Strategies such as blocking endosomal degradation of AAV with proteasome inhibitors significantly improve AAV transduction in mice [148,149]. Switching AAV capsid proteins to an alternative serotype such as AAV8 can also enhance uncoating of the vector and release of the genome [146].

The dsDNA genome of the AAV vector can persist as an episomal element in transduced cells for long periods of time in a variety of molecular forms, including circular monomers, linear monomers and linear concatemers by head to tail recombination of the ITRs. Integration of single and concatemeric genomes into the chromosomal DNA of the host cells occurs at low frequency [103]. Because the transgene is predominantly expressed from the episomal form, expression usually declines over time due to dilution in the replicating cells or degradation in non-dividing cells [105]. A recent study found that administration of Ad 10-20 weeks after AAV gene transfer augmented AAV transgene expression two-fold by increasing the level of pro-viral mRNA [149] and this strategy may prove useful in clinical practice when transgenic protein expression levels fall.

Slow conversion of the virus single stranded (ss) to the double stranded (ds) DNA genome is another issue. After the AAV virus enters the nucleus, the virus single stranded DNA genome (ssDNA) is converted to a transcriptionally active double stranded DNA (dsDNA) [150]. Unless the conversion happens, ssDNA is lost rapidly after transduction, leading to a drop in transgenic protein expression. This rate-limiting conversion process can be circumvented by modifying the configuration of the provirus so that it is packaged as complementary dimer as opposed to the conventional ss [151]. This self-complementary (sc) AAV vector configuration has been shown to significantly improve gene transfer to the liver for human factor IX, achieving levels of stable transduction that are almost one order of magnitude higher than those achieved with an equivalent dose of comparable ssAAV [151,152]. Lowering the required dose of scAAV vector would be of benefit for safety considerations and for scaling up to clinical grade vector production. Modifying the promoter can alter the tissue-specific expression. Use of the liver-specific promoter, LP-1 for example in a self-complementary AAV2/3 vector driving the human factor IX (hFIX) protein, resulted in transgenic h(FIX) protein expression confined to the liver as detected by RT-PCR analysis [152]. This would be beneficial for FH gene therapy.

One of the earliest studies on AAV vectors for FH gene therapy found promising results. Reversal of hypercholesterolaemia was demonstrated in LDLR-/- mice fed with a high cholesterol diet after intraportal vascular injection of 1 × 10^12 ^AAV-2 vector particles encoding the murine VLDLR driven by the CMV enhanced chicken β-actin promoter [153]. Western blot analysis and immunohistochemistry revealed high levels of VLDLR expression in approximately 2-5% of cells of liver harvested at 3 and 6 months after vector delivery with a low vector DNA copy number of 1 copy/cell. Serum cholesterol progressively declined after vector administration and by 6 months, the aortic atherosclerotic lesion area was reduced 33% compared with control mice injected with saline. Phenotypic correction was incomplete however, primarily due to immune activation by the vector products and low efficiency of gene transfer mediated by AAV-2.

Lebherz and colleagues [154] compared the efficiency of AAV-2, -7 and -8 serotype vectors carrying the human LDLRcDNA expressed from a liver specific promoter based on the human thyroxin binding globulin [155]. A vector dose of 1 × 10^12 ^genome copies (gc) per mouse was injected into the portal veins of LDLR-/- mice that were fed a high-fat diet. Transduction efficiency was increased to 50 gc/cell and 10 gc/cell after treatment with an AAV-8 or AAV-7 vector respectively, compared with 2 gc/cell after administration of an AAV-2 vector. Animals receiving the AAV-LDLR serotype 7 and 8 achieved nearly complete normalization of serum lipids and failed to develop the severe atherosclerosis that characterized the untreated animals, with no apparent toxicity observed. Animals treated with the AAV-2 vector achieved partial lipid correction and only a modest improvement in atherosclerosis. Serotype 8 virus achieved stable transduction and expression of the transgene in up to 85% of the hepatocytes. These results are encouraging especially since no expression-terminating immune responses were detected [154]. There were similar findings in the apo-E mouse model of FH, where intravenous administration of AAV2/7- and AAV2/8-apoE vectors completely prevented atherosclerosis at 1 year [156].

Another approach using AAV vectors has been to try to counteract the development of atherosclerosis by gene transfer of interleukin-10 (IL10), an anti-inflammatory cytokine. Injection of AAV IL10 vector into the tail vein [157] of LDLR-knockout mice or into the tibial muscle [158] of apo-E deficient mice resulted in significantly lower levels of atherosclerosis.

More recently, a single intravenous injection of an AAV8 vector containing the mouse LDLR gene to a humanized mouse model of FH, the LDLR-/-Apobec-/- mouse, was found to significantly reduce plasma cholesterol and non-HDL cholesterol levels in chow-fed animals at low doses. Treated mice realized an 87% regression of atherosclerotic lesions with substantial remodeling, after 3 months compared to baseline mice [159].

In summary, modifying the AAV vector system by altering the capsid (reviewed in [160]), including dsDNA and using a liver specific promoter may result in long term, stable and liver specific AAV mediated transgenic protein expression which may be suitable for FH gene therapy.

### Lentivirus based vectors

Lentiviruses (LVs) are a complex sub-group of RVs responsible for a variety of immunological and neurological diseases. Their biological and molecular and properties have been used to classify them as lenti-(sIow) retroviruses. They can be subdivided into primate and non-primate viruses. The primate viruses are the human and simian immunodeficiency viruses (HIV and SIV), and the non-primate viruses include the feline and bovine immunodeficiency viruses, the caprine arthritis/encephalitis virus, the visna/maedi/ovine progressive pneumonia virus, and the equine infectious anaemia virus (EIAV) [107]. As for all RVs, the LV genome consists of a positive-strand polyadenylated RNA of about 10 kb and includes three genes; *gag*, *pol*, and *env *organised in the 5' to 3' orientation. Lentiviruses have additional unique small ORFs located between *pol *and *env *at the 3' terminus, which contain genes for regulatory proteins [107].

Interest in LVs as putative gene transfer systems is derived from the fact that they have the potential to integrate efficiently into the genome of dividing and non-dividing cells providing the possibility for lifetime correction with a single administration of vector [161,162]. Unlike the RV pre-integration complex, which can only reach the target cell nucleus when the nuclear membrane is disrupted during mitosis, the lentiviral PIC contains nuclear localisation signals, which mediate their transport through nuclear membrane pores into the nucleus during the cell interphase [163-165].

Although integration of linear DNA episome, provirus precursor, is generally regarded as the end point of gene transfer, two circular episomal types with intact viral coding regions are also generated by cellular proteins from retro- or lenti-viruses and their derived vectors [166]. The first type circularizes by non-homologous recombination of end-joining to form a circular episome with two adjacent LTRs (2-LTR circular episome) [167]. The second type circularizes by homologous recombination within the LTRs to form a circular episome with a single LTR (1-LTR circular episome) [168]. It has been estimated that approximately one-third of linear lentiviral DNA become circular episomal forms and can express proteins and remain metabolically stable and transcriptionally competent in target cells, although, the single LTR circular episomal forms are more prevalent than 2-LTR circles [166].

Using a LV backbone, two types of vector system can be produced and used for gene transfer, the first are integrated lentivirus based vectors (ILV) and the second are integration deficient lentiviral vectors (IDLV). Initial research on the development of lentivirus-based vectors has focused mainly on HIV-1 derived integrated LV vectors as prototype. This is facilitated by the abundance of knowledge that has been accumulated on this virus since its recognition in 1984 as the causative agent of acquired immuno-deficiency syndrome. Like other virally derived vectors, the initial problem to overcome is to maintain viral infectivity but to render the virus replication deficient [161,162]. The LV based vector design is very similar to that of the three-plasmid co-transfection RV system based on MoMLV, described above. In addition, the emergence of a host immune response against lentiviral vectors has not been shown in most of the preclinical studies [169-173].

Due to the increased concern of insertional mutagenesis (IM) caused by integrating retro- and lentivirus based vectors (as will be discussed later), IDLVs has been thought of as a logical alternative to alleviate the risk of IM. IDLV particles can be generated by the use of integrase mutations that specifically prevent proviral integration resulting in the generation of increased levels of circular vector episomes in transduced cells, but not to compromise its other functions, Because these lentiviral circular episomes lack replication signals, they are gradually lost by dilution in the transduced actively dividing cells, but are stable for several months in transduced quiescent cells [174-176]. Compared to integrating lenti-vectors, IDLVs have a significantly reduced risk of causing IM, a lesser risk of generating RCRs, a reduced risk of transgene silencing [177], and also extremely low levels of integration (residual background integration frequencies of IDLVs in cultured cells through non-integrase pathways are within the range described for plasmid transfection (reviewed in [166,178]).

Recent studies using IDLVs have demonstrated effective gene transfer in the eye [176], brain [174,179], muscle [180], and to a lesser extent in the liver [181], albeit at lower expression levels than with integrating vectors. In addition to gene transfer, IDLVs are also proficient vectors for gene repair and can be converted into stable, replicating circular episomes. These properties, combined with their highly reduced risk of causing IM, have led to increased interest on IDLVs for gene transfer and therapy. Because of the possibility of mobilization by superinfection with replication competent viruses, it has been suggested that future IDL-based vectors should carry *att *mutations in addition to those in the integrase to minimize integration in the event of vector mobilization (reviewed in [166,178]). Long-term evidence for lack of genomic integration beyond residual levels warrants future investigation. To date, IDLVs have not been used for LDLR gene transfer and FH gene therapy.

Many labs including ours [182] experienced difficulties to produce infectious ILVs for transfer and expression of human LDLR under control of a ubiquitous promoter. However, based on the utilisation of a previously characterised liver specific promoter (LSP) [183], Kankkonen and colleagues were able to demonstrate for the first time the successful construction and production of high titre (1 × 10^9 ^IU) third-generation HIV-1 based lentiviral vectors encoding rabbit LDLR. LSP-driven transgene expression was detected after *in vitro *gene transfer into human hepatoma (HepG2) cells, but not after transfer into HeLa cells, HEK 293 cells, or WHHL rabbit skin fibroblasts [183,184]. *In vivo *injection of 1 × 10^9 ^infectious virus particles into the portal vein of WHHL rabbits resulted in liver-specific expression of the LDLR and clinical chemistry and histological analyses showed normal liver function and morphology during the 2-year follow-up without safety issues. This vector dose resulted in low transduction efficiency (<0.01%) but demonstrated on average a147 ± 7% decrease in serum cholesterol levels during the first 4 weeks, a 44 ± 8% decrease at 1 year and a 34 ± 10% decrease at the 2-year time point, compared to the control rabbits infected with HIV-green fluorescent protein. During this period, 70% of the rabbits treated with the liver specific lentiviral LDLR vector demonstrated a positive treatment effect with lowered plasma cholesterol levels (25 ± 8%). However, the detailed pattern of bio-distribution after HIV-vector mediated gene transfer, to evaluate potential risks for possible IM and germ-line transmission, has not been investigated.

### Vector safety in gene therapy

The integration of RV and LV into the genome during gene therapy has caused concern because of the potential for vector-related deleterious side effects on the host. This is, in part, due to the fact that vector insertion occurs in a semi-random manner into actively transcribed genes. For RV vectors insertion preference is for gene promoter regions [185-188] whereas LVs appear to target the transcription unit of the gene [189,190] and therefore are believed less likely to cause effects on host gene expression following integration [191-194]. Genotoxicity by RV vectors associated with insertional mutagenessis (IM) has been studied for several years and the theoretical calculated estimates of mutagenesis at a haploid locus are supported by *in vitro *studies using model systems based on mutagenesis of the *hprt *locus or genes that control promotion of growth factor independence at frequencies between 10^-5^-10^-7 ^per provirus insertion [191,192]. Hence, the likelihood of adverse events caused by RV integration following therapeutic application was considered remote. Unfortunately and unexpectedly, however, development of clonal dominance has been observed in two patient trials that is attributed to RV mediated IM [86,195-199].

In an *ex vivo *trial carried out in France that used patients' own haematopoietic stem cells for transplantation after retroviral transduction to correct X-linked severe combined immuno-deficiency (X-SCID), clonally dominant clones have developed into leukaemias in 4 of these patients [85,86,196]. This also occurred in one patient in a British X-SCID trial [198]. In 4 of these cases integration by MoMLV is believed to have caused IM by inserting near the *LMO2 *gene [196,198]. In addition, insertions have been found in both *BMI1 *and *CCND2 *proto-oncogenes [196,198]. Although 5 out of the 20 patients that enrolled in the French and British trials have developed leukaemia it is difficult to understand clearly the events leading to this disease because of existing genetic abnormalities in the patients' cells that have also been identified. These include chromosomal translocations, gain-of-function mutations activating *NOTCH1*, deletion of tumour suppressor gene *CDKN2A*, 6q interstitial losses, and *SIL-TAL1 *rearrangement [196,198].

In a more recent trial for chronic granulomatous disease (CGD) clonal dominance has also been attributed to retrovirus mediated IM 5 month after vector delivery in 2 patients [199]. Vector integrations activated the zinc finger transcription factor homolog's MDS1/EVI1, PRDM16 or SETBP1 raising concerns that this could eventually cause tumourgenesis. The first affected patient died 2.5 years after vector delivery as a result of a severe sepsis and the second patient has undergone allogeneic transplant [199,200].

In response to these findings, *ex-vivo *and *in vitro *models have been developed in order to examine RV and LV genotoxicity using haematopoietic cells. *Ex vivo *gene therapy using stem cells is considered a more controllable way of introducing genetic modification to the host than by direct systemic vector administration *in vivo *[201-205]. These models have confirmed that insertion of RV, and to a lesser extent SIN-RV and LV can contribute to leukaemic development [85,201-206]. Factors implicated in this process include the integrated vector copy number, integration sites, vector configuration and even the transgene carried by the vector [85,201-206]. Most recently, host cell transcription, in combination with the mutational potential of the vector, has been shown to be involved in the emergence of clonal dominance [206,207].

In our laboratory we have developed a model more suited to gene therapy for FH where vectors may be delivered directly *in vivo. *In this model vector application *in utero *is performed via the fetal mouse circulation that results in gene transfer to most organs, although the liver is mainly transduced [208]. We found that using a primate HIV-1 based vector carrying the human factor IX (hFIX) gene to correct haemophilia in a knockout mouse model of this disease comprehensive cure was achieved without adverse effects, however, the use of a non-primate EIAV vector driving hFIX gene expression led to a high frequency of liver tumours in these mice [209]. This model is still under development, and we have also obtained similar results with the non-primate feline immuno-defificiency (FIV) vector (Themis *et al*. unpublished data). Most importantly in these tumours, we find insertions within genes assigned as candidate genes involved in cancer development (within a 100 kb integration site window - the theoretical distance by which vector insertion is believed to influence expression of a gene carrying the integrated vector). More than 50% of these genes are registered in the Mouse Retroviral Tagged Cancer Gene Database (RTCGD) [210]. Furthermore, many genes carrying insertions have altered gene expression suggestive of IM by the non-primate LV. Hence, using *in utero *gene delivery where genes are in a highly active transcription state, we are able to sensitively detect adverse effects caused by vector integration.

The current models for vector associated genotoxicity all rely on the use of rodent cells as a measure of IM. As these cells are more predisposed to tumour development than human cells, each must be viewed with caution as reliable predictors for mutagenesis occurring in the clinic. The finding of vector genotoxicity in the clinic has, however, revived the use of models of genotoxicity to obtain useful information regarding safe vector design. They may also help to elucidate possible mechanisms relating to IM. In summary, the importance of genotoxicity assays to understand the cause and measure the risk of adverse effects by gene therapy of FH and indeed the treatment of any disease with these vectors cannot be overstated. With the current genotoxicity assays in place we are becoming more confident that gene therapy to FH homozygotes will be possible with minimal side effects.

## Conclusion

Several novel therapies have been developed recently to lower LDL-C in homozygous and heterozygous FH patients [57-65,211]. However, their major drawback is the need for life-long repeated administration in a similar manner to conventional pharmacological drugs. The advantage of gene therapeutic intervention over other therapeutic regimes is the potential for lifetime correction with a single vector administration. Yet, this goal still needs to be achieved. Despite the considerable progress, made in optimising the two most commonly used gene therapy vector-groups based on retro- and adenoviruses, neither vector has been found to be ideal for *in vivo *and/or *ex vivo *gene transfer. Vectors derived from AAV and LVs are very promising. However, the oncogenesis risk from semi-random integration into actively transcribing genes of the host by LVs [189,212,213], possible germline transmission [214] and some immunological reaction after AAV gene transfer in the human haemophilia-B trials [144,215] are critical drawbacks that require further vector development and improvement.

In addition to the LDLR gene augmentation approach, the successful use of the VLDLR as an effective surrogate lipoprotein receptor gene [139-141,153,216,217] for the complementation of mutated LDLR function in homozygote FH patients would also open an alternative therapeutic avenue, since it would avoid the immune problem in patients with no natural LDLR. Despite the fact that most of the pre-clinical and clinical studies were aimed at treatment of the homozygous form of FH, a minority of heterozygous FH patients, who are refractory to existing pharmacological therapy, are also possible targets. Therefore, once a safe and efficient transfer vector is developed and shown to be effective in homozygous FH, its application might be extended to severe heterozygous FH as well. Clearly there is also an urgent demand for safe and efficient vectors that would integrate into the host genome and provide long-term appropriate gene expression for *in vivo *and/or *ex vivo *gene therapy of FH and many other human diseases.

Future work will generally focus on making gene transfer vectors safer by improving their immunogenic, integration, expression and targeting profile. Reducing the inherent oncogenic danger of integrating vectors by engineering conditional suicide genes into the vector backbone to provide a self-destructive mechanism in case of oncogenesis or by targeting their integration into specific pre-defined benign genomic sites, i.e. by zinc-finger nuclease technology, may help achieving this goal [218]. In combinations with the above strategies, the use of *ex vivo *transduction to reduce vector spread can also improve the safety outcome, particularly, if autologous or induced pluripotent stem cells are the target. The application of viral or non-viral, integrating or non-integrating vectors for long term persistence in stem cells with self renewal and differentiation capacities will also be important perspectives for gene-based stem cell therapy.

## Competing interest Disclosure

The authors declare that they have no competing interests.

## Authors' contributions

FAA is responsible for conceiving this work and writing the manuscript. CC, SW, ALD, RH, and MT participated in the drafting of this manuscript. All authors read and approved the final manuscript.

## Author's information

F. A. A. is an Assistant Professor of Genetics and Molecular Medicine, Department of Medical Genetics, Faculty of Medicine, Umm Al-Qura University, Al-Abedia Campus, Makkah 21955, Saudi Arabia. C. C. is an Emeritus Professor and Former Leader, Gene Therapy Research Group, Department of Molecular and Cell Medicine, Sir Alexander Fleming Building, Faculty of Medicine, Imperial College London, London SW7 2AZ, UK. S. W. is a Lecturer and Group Leader, Prenatal Gene Therapy Research Group, Department of Haematology, Haemophilia Centre and Haemostasis Unit, Royal Free and University College Medical School, London NW3 2PF, UK. R. H. is a Research Fellow and Gene Therapy Group Leader in the Section of Molecular Medicine in the Sir Alexander Fleming Building, NHLI, Imperial College London, London SW7 2AZ, UK. A. L. D. is a Senior Lecturer and Honorary Consultant in Obstetrics and Maternal/Fetal Medicine, and leads the Prenatal Cell and Gene Therapy Group, Institute for Women's Health, University College London and UCLH, 86-96 Chenies Mews, London, WC1E 6HX, UK. M. T. is a Lecturer and Group Leader, Gene Therapy and Genotoxicity Research Group, Brunel University, Heinz Wolff Building, Uxbridge, Middlesex, West London UB8 3PH, UK.
